# Supplementary material for: Early Life Factors Influencing Children Gut Microbiota at 3.5 Years from Two French Birth Cohorts
Source: Microorganisms. 2023 May 25;11(6):1390. doi: 10.3390/microorganisms11061390 (PMC10301311; doi:10.3390/microorganisms11061390)
Supplement: Supplementary file 1 [file microorganisms-11-01390-s001.zip › microorganisms-2380861-supplementary.pdf]

Table S1. Characteristics of the study population (n = 798)

	N (%) or Mean (SD) and median (IQR)		
**Maternal Characteristics**	**Pooled sample (N=798)**	**ELFE (N=626)**	**EPIFLORE (N=172)**
**Mother born in France**			
Yes	730 (91.5%)	582 (93.0%)	148 (86.0%)
No	68 (8.5%)	44 (7.0%)	24 (14.0%)
**Maternal age (Years)**			
<25	40 (5.0%)	24 (3.8%)	16 (9.3%)
[25-35[	561 (70.3%)	452 (72.2%)	109 (63.4%)
≥35	188 (23.6%)	150 (24.0%)	38 (22.1%)
Missing	9 (1.1%)	0 (0%)	9 (5.2%)
**Maternal level of education**			
< High School	49 (6.1%)	11 (1.8%)	38 (22.1%)
High school	163 (20.4%)	133 (21.2%)	30 (17.4%)
1-2 years of tertiary education	202 (25.3%)	166 (26.5%)	36 (20.9%)
≥ 3 years of tertiary education	376 (47.1%)	310 (49.5%)	66 (38.4%)
Missing	8 (1.0%)	6 (1.0%)	2 (1.2%)
**Household income**			
<1500€	24 (3.0%)	14 (2.2%)	10 (5.8%)
[1500-4000€[	473 (59.3%)	381 (60.9%)	92 (53.5%)
>=4000€	246 (30.8%)	212 (33.9%)	34 (19.8%)
Missing	55 (6.9%)	19 (3.0%)	36 (20.9%)
**Maternal BMI**			
Underweight	57 (7.1%)	48 (7.7%)	9 (5.2%)
Normal	523 (65.5%)	409 (65.3%)	114 (66.3%)
Overweight	138 (17.3%)	113 (18.1%)	25 (14.5%)
Obese	60 (7.5%)	47 (7.5%)	13 (7.6%)
Missing	20 (2.5%)	9 (1.4%)	11 (6.4%)
**Children Characteristics**			
**Sex**			
Boys	443 (55.5%)	356 (56.9%)	87 (50.6%)
Girls	355 (44.5%)	270 (43.1%)	85 (49.4%)
**Gestational age (weeks)**			
Mean (SD)	37.3 (4.61)	39.6 (1.35)	29.1 (1.97)
Median [IQR]	39.3 [37.00 ; 40.29]	39.7 [39.00 ; 40.57]	29.6 [27.43 ; 30.71]
Missing	13 (1.6%)	13 (2.1%)	0 (0%)
**Delivery mode**			
Vaginal	557 (69.8%)	497 (79.4%)	60 (34.9%)
Cesarean	220 (27.6%)	109 (17.4%)	111 (64.5%)
Missing	21 (2.6%)	20 (3.2%)	1 (0.6%)
**Early antibiotics**			
No	611 (76.6%)	573 (91.5%)	38 (22.1%)
Yes	132 (16.5%)	13 (2.1%)	119 (69.2%)
Missing	55 (6.9%)	40 (6.4%)	15 (8.7%)
**Skin-to-skin practice**			
Yes	676 (84.7%)	573 (91.5%)	103 (59.9%)
No	99 (12.4%)	37 (5.9%)	62 (36.0%)
Missing	23 (2.9%)	16 (2.6%)	7 (4.1%)
**Human milk consumption**			
No	146 (18.3%)	141 (22.5%)	5 (2.9%)
Yes	576 (72.2%)	440 (70.3%)	136 (79.1%)
Missing	76 (9.5%)	45 (7.2%)	31 (18.0%)
**Duration of human milk consumption (in months)**			
Mean (SD)	3.89 (5.08)	3.98 (5.44)	3.51 (3.21)
Median [IQR]	2.46 [0.23 ; 5.98]	2.46 [0.03 ; 6.00]	2.22 [0.92 ; 5.68]
Missing	76 (9.5%)	45 (7.2%)	31 (18.0%)
**Household siblings**			
No	327 (41.0%)	274 (43.8%)	53 (30.8%)
At least 1	439 (55.0%)	345 (55.1%)	94 (54.7%)

Missing	32 (4.0%)	7 (1.1%)	25 (14.5%)
**Daycare exposure**			
Family	261 (32.7%)	171 (27.3%)	90 (52.3%)
Childcare assistant/Paid home help	418 (52.4%)	355 (56.7%)	63 (36.6%)
Daycare centre	106 (13.3%)	87 (13.9%)	19 (11.0%)
Missing	13 (1.6%)	13 (2.1%)	0 (0%)
**Elfe only** (39.6 ± 1.35 weeks of GA)			
**Age at global diversification**			
<=6 months	-	489 (78.1%)	-
>6 months	-	65 (10.4%)	-
Missing	-	72 (11.5%)	-
**Age at first vegetables introduction**			
<=6 months	-	446 (71.2%)	-
>6 months	-	108 (17.3%)	-
Missing	-	72 (11.5%)	-
**Age at first fruits introduction**			
<=6 months	-	413 (66.0%)	-
>6 months	-	142 (22.7%)	-
Missing	-	71 (11.3%)	-
**Age at first meat introduction**			
[4-6 months]	-	111 (17.7%)	-
>6 months	-	433 (69.2%)	-
Missing	-	82 (13.1%)	-
**Age at first fish introduction**			
[4-6 months]	-	79 (12.6%)	-
>6 months	-	458 (73.2%)	-
Missing	-	89 (14.2%)	-
**PNNS-score***			
<= 7.80	-	293 (46.8%)	-
> 7.80	-	296 (47.3%)	-
Missing	-	37 (5.9%)	-
**Pregnancy score***			
<= 7.75	-	303 (48.4%)	-
> 7.75	-	286 (45.7%)	-
Missing	-	37 (5.9%)	-
**Antibiotics in the first year**			
Yes	-	375 (59.9%)	-
No	-	242 (38.7%)	-
Missing	-	9 (1.4%)	-
**Environnement**			
Rural	-	180 (28.8%)	-
Urban	-	439 (70.1%)	-
Missing	-	7 (1.1%)	-
**Epiflore only** (29.1 ± 1.97 weeks of GA)			
**Antibiotics during pregnancy**			
No	-	-	84 (48.8%)
Yes	-	-	86 (50.0%)
Missing	-	-	2 (1.2%)
**Antibiotics during delivery**			
No	-	-	97 (56.4%)
Yes	-	-	53 (30.8%)
Missing	-	-	22 (12.8%)

Continuous variables are given as mean (sd) and median (IQR). Distributions in categorical variable are given in numbers of events (percentage). Missing data are displayed if any.
Abbreviations: BMI, Body Mass Index; PNSS, Programme National Nutrition Santé; IQR, Interquartile range .
*Chosen cut points are median scores within the population.

Table S2. Differential abundance testing results for factors significantly associated the gut microbiota composition in pooled sample according to three different methods

				LDM			ANCOM-BC				ALDEx2		
	otu.id	Genus	Direction	adj.pvalue	mean.freq	beta	adj.pvalue	beta	se	W	adj.pvalue	beta	se
	Gestational age (<37 weeks of GA vs >= 37 weeks of GA)												
	Cluster_117	Butyricicoccus	-	0.0007	0.0021	-0.0086	0.0000	-0.6183	0.1044	-5.9225	0.0001	-0.9918	0.1883
	Cluster_31	Anaerostipes	-	0.0007	0.0003	-0.0024	0.0000	-0.7993	0.1247	-6.4112	0.0000	-1.1673	0.1880
	Cluster_72	[Eubacterium] hallii group	-	0.0007	0.0108	-0.0505	0.0000	-0.8436	0.1454	-5.8010	0.0118	-1.3353	0.3207
	Cluster_8	Blautia	-	0.0007	0.0037	-0.0223	0.0000	-0.5962	0.0910	-6.5490	0.0000	-0.8292	0.1458
	Cluster_299	Erysipelotrichaceae UCG-003	-	0.0007	0.0357	-0.1341	0.0000	-0.6334	0.1013	-6.2540	0.0483	-1.4243	0.3472
	Cluster_71	Ruminococcaceae (unknown genus)	+	0.0053	0.0058	0.0317	0.0170	0.3611	0.1111	3.2493	0.0475	0.5671	0.1576
	Cluster_6	Bifidobacterium	-	0.0148	0.0400	-0.1344	0.0059	-0.6624	0.1794	-3.6931	0.0135	-0.9844	0.2483
	Household siblings (At least 1 vs none)												
	Cluster_108	Dorea	+	0.0005	0.0019	0.0079	0.0001	0.5441	0.1195	4.5521	0.0082	1.0875	0.2569
	Cluster_170	Flavonifractor	-	0.0005	0.0009	-0.0043	0.0000	-0.6606	0.0965	-6.8477	0.0000	-1.1689	0.1882
	Cluster_190	Oscillospiraceae UCG-003	+	0.0005	0.0008	0.0076	0.0000	0.6487	0.1199	5.4091	0.0159	1.3621	0.3127
	Cluster_58	Coprococcus	+	0.0005	0.0075	0.0349	0.0001	0.7745	0.1761	4.3970	0.0245	1.3449	0.3436
	Cluster_62	Odoribacter	+	0.0005	0.0048	0.0169	0.0002	0.6152	0.1446	4.2535	0.0169	1.1253	0.2841
Genus level	Cluster_68	Christensenellaceae R-7 group	+	0.0005	0.0074	0.0711	0.0000	0.9164	0.1496	6.1244	0.0001	1.4919	0.2781
	Cluster_69	Lachnospiraceae ND3007 group	+	0.0005	0.0028	0.0182	0.0000	1.0226	0.1579	6.4762	0.0000	1.9715	0.3498
	Cluster_79	Barnesiella	+	0.0005	0.0072	0.0362	0.0002	0.8071	0.1901	4.2461	0.0369	1.4501	0.3843
	Cluster_82	Oscillospiraceae UCG-002	+	0.0005	0.0069	0.0476	0.0000	0.9410	0.1485	6.3350	0.0000	1.6395	0.2896
	Cluster_240	Eisenbergiella	-	0.0015	0.0008	-0.0040	0.0000	-0.6692	0.1187	-5.6390	0.0002	-1.3905	0.2629
	Cluster_1	Bacteroides	-	0.0027	0.2980	-0.4491	0.0001	-0.3169	0.0687	-4.6133	0.0001	-0.5553	0.1086
	Cluster_389	Eggerthella	-	0.0039	0.0002	-0.0011	0.0000	-0.5411	0.0969	-5.5853	0.0023	-1.2337	0.2582
	Cluster_403	[Ruminococcus] gnavus group	-	0.0046	0.0007	-0.0003	0.0000	-0.7190	0.1279	-5.6224	0.0001	-1.5348	0.2913
	Cluster_8	Blautia	-	0.0092	0.0358	-0.0860	0.0001	-0.3216	0.0728	-4.4162	0.0002	-0.5626	0.1148
	Cluster_109	Oscillibacter	-	0.0115	0.0013	-0.0034	0.0000	-0.4196	0.0846	-4.9626	0.0218	-0.7134	0.1770
	Cluster_186	Ruminococcaceae UBA1819	-	0.0143	0.0005	-0.0015	0.0000	-0.4016	0.0806	-4.9832	0.0057	-0.8045	0.1845
	Cluster_65	Tyzzerella	-	0.0192	0.0043	-0.0108	0.0006	-0.7320	0.1830	-4.0006	0.0449	-0.5762	0.1257
	Cluster_85	Lachnoclostridium	-	0.0318	0.0081	-0.0084	0.0000	-0.3760	0.0763	-4.9292	0.0001	-0.6443	0.1256
	Mother born in France (no vs yes)												
	Cluster_174	Oscillospiraceae (unknown genus)	-	0.0093	0.0011	-0.0036	0.0016	-0.5830	0.1346	-4.3310	0.0284	-0.2904	0.1426
	otu.id	Family											
	Gestational age (<37 weeks of GA vs >= 37 weeks of GA)												
	Cluster_117	Butyricicoccaceae	-	0.0006	0.0021	-0.0086	0.0003	-0.4427	0.1062	-4.1684	0.0000	-1.0260	0.1934
	Cluster_299	Erysipelatoclostridiaceae	-	0.0006	0.0004	-0.0026	0.0000	-0.5645	0.1027	-5.4944	0.0001	-1.6502	0.2912
	Cluster_82	Oscillospiraceae	+	0.0025	0.0151	0.0454	0.0000	0.4425	0.0632	7.0015	0.0178	0.3947	0.1077
	Cluster_6	Bifidobacteriaceae	-	0.0082	0.0400	-0.1343	0.0350	-0.4868	0.1771	-2.7486	0.0027	-1.0159	0.2449
	Household siblings (At least 1 vs none)												
Family level	Cluster_62	Marinifilaceae	+	0.0004	0.0054	0.0188	0.0396	0.3352	0.1318	2.5443	0.0235	0.9008	0.2537
	Cluster_68	Christensenellaceae	+	0.0004	0.0075	0.0710	0.0000	0.7037	0.1483	4.7464	0.0001	1.4045	0.2785
	Cluster_79	Barnesiellaceae	+	0.0004	0.0081	0.0364	0.0084	0.5600	0.1802	3.1083	0.0220	1.2976	0.3600
	Cluster_1	Bacteroidaceae	-	0.0025	0.2980	-0.4494	0.0000	-0.4985	0.0702	-7.1050	0.0000	-0.5693	0.1091
	Cluster_25	Veillonellaceae	-	0.0324	0.0081	-0.0190	0.0000	-0.9436	0.2070	-4.5573	0.0355	-1.4105	0.4026
	Cluster_389	Eggerthellaceae	-	0.0457	0.0004	-0.0007	0.0000	-0.5609	0.0811	-6.9158	0.0050	-0.8047	0.1886
	Cluster_6	Bifidobacteriaceae	-	0.0457	0.0398	-0.1214	0.0000	-0.5517	0.1225	-4.5047	0.0262	-0.6623	0.1925

LDM: mean.freq= the mean relative abundance of taxa in the tested category, beta= effect size. ANCOM-BC: beta= coefficient obtained from the ANCOM-BC log linear (natural log) model (log-transformed change in abundance), se = standard error of the beta, W = test statistic (beta/se). ALDEx2: beta= per-feature effect size, se= standard error of the beta. Direction: - = less abundant taxa in the category compared to the reference, + = more abundant taxa in the category compared to the reference. adj.pvalue = False discovery rate (FDR) adjusted p-value.

Table S3. Multivariate association between children enterotypes and early life factors in ELFE children

		Enterotype P_type vs B_type		
	**N**	**OR[a]**	**95% CI**	**p-value**
**Gestational age (weeks)**	588	1.00	0.86, 1.18	0.95
**Delivery mode**	578			0.45
Vaginal		—	—	
Cesarean		0.80	0.43, 1.41	
**Early neonatal antibiotics**	557			0.89
No		—	—	
Yes		0.89	0.12, 3.99	
**Human milk consumption**	537			**0.033**
No		—	—	
Yes		1.87	1.05, 3.50	
**Duration of human milk consumption (months)**	537	1.01	0.96, 1.05	0.72
**Skin-to-skin practice**				0.63
Yes		—	—	
No		1.23	0.50, 2.71	
**Maternal prepregnancy BMI**	617			0.63
Underweight		1.09	0.48, 2.27	
Normal		—	—	
Overweight		1.01	0.57, 1.72	
Obese		1.62	0.77, 3.19	
**Daycare exposure**	598			**0.011**
Family		—	—	
Childcare assistant/Paid home help		0.54	0.33, 0.90	
Daycare centre		1.14	0.58, 2.19	
**Household siblings**	604			0.062
No		—	—	
At least 1		1.53	0.98, 2.42	
**Mother born in France**	625			0.66
Yes		—	—	
No		0.83	0.33, 1.80	

Logistic regression models.

Abbreviations: N = number of children included in each model; P_type, enterotype enriched in *Prevotella*; B_type, enterotype enriched in *Bacteroides*; CI, Confidence Interval; BMI, Body Mass Index

[a] Each covariate is adjusted for its own set of parsimonious confounding factors presented in the "Exposures of interest" section.

*Chosen cut points are median scores within the population

p-values marked with bold indicate statistically significant p-values ($p \leq 0.05$)

Table S4. Multivariate association between alpha diversity metrics and early life factors in ELFE children

		Chao1 estimate			Shannon index		
	**N**	**Beta[a]**	**95% CI**	**p-value**	**Beta[a]**	**95% CI**	**p-value**
**Gestational age (weeks)**	588	3.2	-1.5, 8.0	0.18	0.01	-0.02, 0.03	0.62
**Delivery mode**	578			**0.050**			**0.007**
Vaginal		—	—		—	—	
Cesarean		-17	-34, 0.00		0.12	-0.20, -0.03	
**Early neonatal antibiotics**	557			0.56			0.21
No		—	—		—	—	
Yes		15	-36, 66		0.16	-0.09, 0.41	
**Human milk consumption**	537			0.70			0.61
No		—	—		—	—	
Yes		3.1	-12, 19		0.02	-0.10, 0.06	
**Duration of human milk consumption (months)**	537	0.67	-0.57, 1.9	0.29	0.01	0.00, 0.01	0.055
**Skin-to-skin practice**	557			0.80			0.83
Yes		—	—		—	—	
No		-3.4	-30, 23		0.01	-0.15, 0.12	
**Maternal prepregnancy BMI**	617			0.077			0.091
Underweight		5.9	-18, 30		0.08	-0.03, 0.20	
Normal		—	—		—	—	
Overweight		18	2.0, 35		0.09	0.01, 0.17	
Obese		21	-2.9, 45		0.06	-0.06, 0.18	
**Daycare exposure**	598			0.25			0.53
Family		—	—		—	—	
Childcare assistant/Paid home help		-12	-28, 2.9		0.04	-0.11, 0.04	
Daycare centre		-14	-35, 7.7		0.05	-0.16, 0.05	
**Household siblings**	604			**<0.001**			**<0.001**
No		—	—		—	—	
At least 1		42	29, 54		0.18	0.11, 0.24	
**Mother born in France**	625			0.16			0.20
Yes		—	—		—	—	
No		-17	-41, 6.9		0.08	-0.20, 0.04	

Linear regression models.
Abbreviations: N = number of children included in each model; CI, Confidence Interval; BMI, Body Mass Index
[a] Each covariate is adjusted for its own set of parsimonious confounding factors presented in the "Exposures of interest" section.
*Chosen cut points are median scores within the population
p-values marked with bold indicate statistically significant p-values ($p \leq 0.05$)

Table S5. Association between beta diversity metrics and early life factors in ELFE children

	**Bray Curtis**		**UniFrac**	
**Exposures**	R	P-value	R	P-value
Gestational age (weeks)	0.002	0.643	0.002	0.603
Delivery mode	0.002	0.117	0.002	0.156
Early neonatal antibiotics	0.001	0.809	0.001	0.798
Human milk consumption	0.002	0.627	0.002	0.627
Skin-to-skin pratice	0.001	0.947	0.001	0.962
Maternal prepregnancy BMI	0.006	0.208	0.006	0.168
Daycare exposure	0.004	0.612	0.004	0.608
Household siblings	0.008	**0.005**	0.008	**0.005**
Mother born in France	0.001	0.541	0.001	0.550

Each covariate is adjusted for its own set of parsimonious confounding factors presented in the "Exposures of interest" section. FDR p adjusted ≤ 0.05 are marked in bold.

Table S6. Differential abundance testing results for factors significantly associated the gut microbiota composition according to three different methods in ELFE children

			LDM			ANCOM-BC				ALDEx2		
otu.id	Genus	Direction	adj,pvalue	mean,freq	beta	adj,pvalue	beta	se	W	adj,pvalue	beta	se
Household siblings (At least 1 vs none)												
Cluster_170	Flavonifractor	-	0,0004	0,0008	-0,0043	0,0000	-0,6606	0,0965	-6,8477	0,0000	-1,1187	0,2087
Cluster_190	Oscillospiraceae UCG-003	+	0,0004	0,0007	0,0076	0,0000	0,6487	0,1199	5,4091	0,0050	1,5918	0,3455
Cluster_240	Eisenbergiella	-	0,0004	0,0007	-0,0040	0,0000	-0,6692	0,1187	-5,6390	0,0002	-1,5268	0,2868
Cluster_38	Subdoligranulum	+	0,0004	0,0147	-0,0220	0,0000	0,8376	0,1710	4,8978	0,0001	1,6205	0,3192
Cluster_389	Eggerthella	-	0,0004	0,0002	-0,1213	0,0000	-0,5411	0,0969	-5,5853	0,0002	-1,4653	0,2833
Cluster_58	Coprococcus	+	0,0004	0,0081	0,0349	0,0002	0,7745	0,1761	4,3970	0,0200	1,4785	0,3805
Cluster_62	Odoribacter	+	0,0004	0,0048	0,0169	0,0000	-0,3760	0,0763	-4,9292	0,0041	1,3302	0,3108
Cluster_68	Christensenellaceae R-7 group	+	0,0004	0,0071	0,0148	0,0000	0,9164	0,1496	6,1244	0,0001	1,5802	0,3091
Cluster_69	Lachnospiraceae ND3007 group	+	0,0004	0,0030	0,0182	0,0000	1,0226	0,1579	6,4762	0,0010	1,8642	0,3913
Cluster_82	Oscillospiraceae UCG-002	+	0,0004	0,0065	0,0476	0,0000	0,9410	0,1485	6,3350	0,0000	1,7488	0,3209
Cluster_1	Bacteroides	-	0,0105	0,2910	-0,4491	0,0005	-0,3169	0,0687	-4,6133	0,0005	-0,5559	0,1193
Cluster_85	Lachnoclostridium	-	0,0189	0,0079	-0,0084	0,0000	-0,3760	0,0763	-4,9292	0,0001	-0,6981	0,1398
Cluster_186	Ruminococcaceae UBA1819	-	0,0226	0,0004	-0,0001	0,0000	-0,4016	0,0806	-4,9832	0,0257	-0,8218	0,2030
Cluster_403	[Ruminococcus] gnavus group	+	0,0226	0,0007	-0,0003	0,0000	-0,7190	0,1279	-5,6224	0,0021	-1,5175	0,3276
Cluster_8	Blautia	-	0,0226	0,0390	-0,0860	0,0005	-0,3169	0,0687	-4,6133	0,0012	-0,5508	0,1234
Household siblings (At least 1 vs none)												
otu.id	Family											
Cluster_62	Marinifilaceae	+	0,0006	0,0053	0,0019	0,0122	0,4342	0,1431	3,0344	0,0066	1,0668	0,2717
Cluster_68	Christensenellaceae	+	0,0006	0,0072	0,0079	0,0000	0,7615	0,1662	4,5813	0,0002	1,5174	0,3087
Cluster_1	Bacteroidaceae	-	0,0107	0,2910	-0,0160	0,0000	-0,4831	0,0781	-6,1844	0,0002	-0,5707	0,1213
Cluster_389	Eggerthellaceae	-	0,0107	0,0004	0,0349	0,0000	-0,6280	0,0884	-7,1076	0,0010	-0,9263	0,1983
Cluster_67	Enterobacteriaceae	-	0,0169	0,0027	0,0014	0,0000	-0,7444	0,1559	-4,7749	0,0067	-1,1937	0,3040
Cluster_6	Bifidobacteriaceae	-	0,0348	0,0415	0,0050	0,0000	-0,5820	0,1282	-4,5397	0,0150	-0,7145	0,1988

LDM: mean.freq= the mean relative abundance of taxa in the tested category, beta= effect size. ANCOM-BC: beta= coefficient obtained from the ANCOM-BC log linear (natural log) model (log-transformed change in abundance), se = standard error of the beta, W = test statistic (beta/se). ALDEx2: beta= per-feature effect size, se= standard error of the beta. Direction: - = less abundant taxa in the category compared to the reference, + = more abundant taxa in the category compared to the reference. adj.pvalue = False discovery rate (DFR) adjusted p-value.

Table S7. Multivariate association between pooled sample enterotypes and early life factors with imputed dataset

		Enterotype P_type vs B_type		
	N	OR[a]	95% CI	p-value
**Pooled sample**				
**Gestational age (weeks)**	798	1.02	0.98, 1.07	0.30
**Delivery mode**	798			0.47
Vaginal		—	—	
Cesarean		0.85	0.53, 1.33	
**Early neonatal antibiotics**	798			0.55
No		—	—	
Yes		0.80	0.37, 1.67	
**Human milk consumption**	798			**0.010**
No		—	—	
Yes		2.01	1.17, 3.63	
**Duration of human milk consumption (months)**	798	1.01	0.97, 1.05	0.56
**Skin-to-skin practice**	798			0.32
Yes		—	—	
No		0.73	0.38, 1.35	
**Maternal prepregnancy BMI**	798			0.28
Underweight		0.88	0.39, 1.77	
Normal		—	—	
Overweight		1.16	0.72, 1.84	
Obese		1.80	0.96, 3.23	
**Daycare exposure**	798			**0.001**
Family		—	—	
Childcare assistant/Paid home help		0.78	0.50, 1.22	
Daycare centre		2.11	1.21, 3.66	
**Household siblings**	798			0.082
No		—	—	
At least 1		1.41	0.96, 2.09	
**Mother born in France**	798			0.46
Yes		—	—	
No		1.26	0.67, 2.25	
**ELFE**				
**Age at complementary feeding**	626			0.58
≤ 6 months		—	—	
>6 months		0.03	-0.07, 0.13	
**Age at first vegetables introduction**	626			0.56
≤6 months		—	—	
>6 months		0.85	0.48, 1.45	
**Age at first fruits introduction**	626			0.40
≤ 6 months		—	—	
>6 months		0.80	0.48, 1.32	
**Age at first meat introduction**	626			0.39
≤ 6 months		—	—	
>6 months		0.79	0.47, 1.38	
**Age at first fish introduction**	626			0.20
≤ 6 months		—	—	
>6 months		0.68	0.38, 1.25	
**PNNS score***	626			0.065
≤ 7.80		—	—	
> 7.80		1.49	0.98, 2.29	
**Pregnancy score***	626			0.25
≤ 7.75		—	—	
> 7.75		1.29	0.84, 2.01	

	N			p-value
**Antibiotics in the first year**	626			0.18
Yes		—	—	
No		0.74	0.47, 1.15	
**Environnement**	626			0.55
Rural		—	—	
Urban		1.15	0.73, 1.86	
**EPIPAGE 2**				
**Antibiotics during delivery**	172			0.071
No		—	—	
Yes		0.41	0.15, 1.08	
**Antibiotics during pregnancy**	172			0.55
No		—	—	
Yes		1.31	0.54, 3.11	

Logistic regression models.
Abbreviations: N = number of children included in each model; P_type, enterotype enriched in *Prevotella*; B_type, enterotype enriched in *Bacteroides*; CI, Confidence Interval; BMI, Body Mass Index; PNSS, Programme National Nutrition Santé.
[a] Each covariate is adjusted for its own set of parsimonious confounding factors presented in the "Exposures of interest" section.
*****Chosen cut points are median scores within the population
p-values marked with bold indicate statistically significant p-values ($p \leq 0.05$)

Table S8. Multivariate association between alpha diversity metrics and early life factors with imputed dataset in pooled sample.

		Chao1 estimate			Shannon index		
**Pooled sample**	**N**	**Beta[a]**	**95% CI**	**p-value**	**Beta[a]**	**95% CI**	**p-value**
**Gestational age (weeks)**	798	2.1	0.82, 3.4	**0.001**	0.01	0.00, 0.02	**0.006**
**Delivery mode**	798			**0.004**			**<0.001**
Vaginal		—	—		—	—	
Cesarean		-21	-34, -6.6		-0.11	-0.18, -0.05	
**Early neonatal antibiotics**	798			0.23			0.052
No		—	—		—	—	
Yes		14	-9.1, 38		0.11	0.00, 0.23	
**Human milk consumption**	798			0.28			0.94
No		—	—		—	—	
Yes		8.0	-6.6, 23		0.00	-0.07, 0.07	
**Duration of human milk consumption (months)**	798	1.0	-0.18, 2.1	0.10	0.01	0.00, 0.01	**0.024**
**Skin-to-skin practice**	798			0.63			0.19
Yes		—	—		—	—	
No		4.7	-14, 24		0.06	-0.03, 0.15	
**Maternal prepregnancy BMI**	798			**0.016**			**0.029**
Underweight		-1.0	-23, 21		0.05	-0.06, 0.16	
Normal		—	—		—	—	
Overweight		21	5.6, 36		0.10	0.03, 0.18	
Obese		22	0.70, 44		0.08	-0.02, 0.19	
**Daycare exposure**	798			0.96			0.74
Family		—	—		—	—	
Childcare assistant/Paid home help		1.6	-12, 15		0.02	-0.04, 0.09	
Daycare centre		0.01	-19, 19		0.01	-0.09, 0.10	
**Household siblings**	798			**<0.001**			**<0.001**
No		—	—		—	—	
At least 1		39	27, 50		0.17	0.12, 0.23	
**Mother born in France**	798			0.24			0.057

Yes		—	—		—	—	
No		-12	-32, 8.2		-0.10	-0.19, 0.00	
**ELFE**							
**Age at complementary feeding**	626			0.89			0.58
≤ 6 months		—	—		—	—	
>6 months		1.5	-19, 22		0.03	-0.07, 0.13	
**Age at first vegetables introduction**	626			0.77			0.39
≤6 months		—	—		—	—	
>6 months		2.4	-13, 18		0.04	-0.04, 0.11	
**Age at first fruits introduction**	626			0.88			0.93
≤ 6 months		—	—		—	—	
>6 months		1.1	-13, 15		0.00	-0.07, 0.07	
**Age at first meat introduction**	626			0.95			0.59
≤ 6 months		—	—		—	—	
>6 months		-0.47	-16, 16		0.02	-0.06, 0.10	
**Age at first fish introduction**	626			0.99			0.88
≤ 6 months		—	—		—	—	
>6 months		0.13	-18, 18		-0.01	-0.10, 0.08	
**Maternal pregnancy diet (PNNS score)***	626			0.48			0.67
≤ 7.80		—	—		—	—	
> 7.80		4.4	-7.8, 17		0.01	-0.05, 0.07	
**Maternal pregnancy diet (Pregnancy score)***	626			0.79			0.80
≤ 7.75		—	—		—	—	
> 7.75		1.7	-11, 14		0.01	-0.05, 0.07	
**Antibiotics in the first year**	626			0.36			0.11
Yes		—	—		—	—	
No		5.9	-6.7, 18		0.05	-0.01, 0.11	
**Environnement**	626			0.62			0.81
Rural		—	—		—	—	
Urban		-3.5	-17, 10		-0.01	-0.08, 0.06	
**EPIPAGE 2**							
**Antibiotics during delivery**	172			0.24			0.51
No		—	—		—	—	
Yes		20	-13, 52		0.05	-0.10, 0.20	
**Antibiotics during pregnancy**	172			0.79			0.26
No		—	—		—	—	
Yes		4.2	-27, 36		0.08	-0.06, 0.23	

Linear regression models.
Abbreviations: N = number of children included in each model; CI, Confidence Interval; BMI, Body Mass Index; PNSS, Programme National Nutrition Santé.
[a] Each covariate is adjusted for its own set of parsimonious confounding factors presented in the “Exposures of interest” section.
*Chosen cut points are median scores within the population
p-values marked with bold indicate statistically significant p-values ($p \leq 0.05$)

Table S9. PERMANOVA association between beta diversity metrics and early life factors in pooled sample with imputed dataset

		Bray-Curtis		Unifrac	
**Group**	**Exposures**	R	P-value	R	P-value
Pooled sample	Gestational age	0.006	**0.005**	0.007	**0.005**
Pooled sample	Delivery mode	0.002	0.074	0.002	**0.018**
Pooled sample	Early neonatal antibiotics	0.001	0.525	0.002	0.181
Pooled sample	Human milk consumption	0.001	0.450	0.002	0.161
Pooled sample	Skin-to-skin pratice	0.001	0.955	0.001	0.946
Pooled sample	Maternal prepregnancy BMI	0.005	0.107	0.005	**0.023**
Pooled sample	Daycare exposure	0.004	0.066	0.003	0.296
Pooled sample	Household siblings	0.007	**0.005**	0.012	**0.005**
Pooled sample	Mother born in France	0.002	0.115	0.003	**0.002**
ELFE	Complementary feeding	0.001	0.757	0.002	0.416
ELFE	Vegetables introduction	0.002	0.354	0.003	0.126
ELFE	Fruits introduction	0.003	**0.023**	0.002	0.248
ELFE	Meat introduction	0.002	0.410	0.002	0.425
ELFE	Fish introduction	0.002	0.427	0.002	0.255
ELFE	Maternal pregnancy diet (PNSS score)	0.001	0.740	0.002	0.242
ELFE	Maternal pregnancy diet (Pregnancy score)	0.001	0.919	0.001	0.978
ELFE	Environment	0.001	0.789	0.001	0.635
ELFE	Antibiotics during first year of life	0.002	0.645	0.001	0.648
EPIPAGE 2	Antibiotics during pregnancy	0.004	0.882	0.005	0.672
EPIPAGE 2	Antibiotics during delivery	0.006	0.814	0.006	0.672

Each covariate is adjusted for its own set of parsimonious confounding factors presented in the "Exposures of interest" section. FDR p adjusted ≤ 0.05 are marked in bold.
Abbreviations: BMI, Body Mass Index; PNSS, Programme National Nutrition Santé.

Table S10. Differential abundance testing results for factors significantly associated the gut microbiota composition according to three different methods in pooled sample.

				LDM			ANCOM-BC				ALDEx2		
	otu.id	Genus	Direction	adj.pvalue	mean.freq	beta	adj.pvalue	beta	se	W	adj.pvalue	beta	se
	Gestational age (<37 weeks of GA vs >= 37 weeks of GA)												
	Cluster_117	Butyricicoccus	-	0.001	0.002	-0.010	0.000	-0.640	0.096	-6.658	0.000	-0.958	0.171
	Cluster_299	Erysipelotrichaceae UCG-003	-	0.001	0.000	-0.003	0.000	-0.665	0.088	-7.550	0.017	-1.446	0.302
	Cluster_31	Anaerostipes	-	0.001	0.011	-0.049	0.000	-0.752	0.110	-6.844	0.000	-1.002	0.167
	Cluster_71	Ruminococcaceae (unknown genus)	+	0.001	0.006	0.033	0.017	0.288	0.098	2.933	0.035	0.528	0.141
	Cluster_72	[Eubacterium] hallii group	-	0.001	0.004	-0.028	0.000	-0.949	0.128	-7.418	0.000	-1.475	0.285
	Cluster_8	Blautia	-	0.001	0.035	-0.175	0.000	-0.686	0.077	-8.889	0.000	-0.876	0.126
	Household siblings (At least 1 vs none)												
	Cluster_108	Dorea	+	0.000	0.002	0.008	0.000	0.494	0.114	4.329	0.022	0.956	0.242
	Cluster_170	Flavonifractor	-	0.000	0.001	-0.004	0.000	-0.642	0.092	-6.948	0.000	-1.149	0.179
	Cluster_190	Oscillospiraceae UCG-003	+	0.000	0.001	0.007	0.000	0.640	0.114	5.604	0.006	1.316	0.296
	Cluster_240	Eisenbergiella	-	0.000	0.001	-0.005	0.000	-0.675	0.112	-6.019	0.000	-1.380	0.249
	Cluster_389	Eggerthella	-	0.000	0.000	-0.001	0.000	-0.535	0.093	-5.727	0.001	-1.212	0.245
Genus level	Cluster_58	Coprococcus	+	0.000	0.007	0.039	0.000	0.782	0.168	4.653	0.015	1.330	0.326
	Cluster_62	Odoribacter	+	0.000	0.005	0.019	0.000	0.641	0.138	4.641	0.004	1.178	0.270
	Cluster_68	Christensenellaceae R-7 group	+	0.000	0.008	0.070	0.000	0.935	0.142	6.565	0.000	1.499	0.266
	Cluster_69	Lachnospiraceae ND3007 group	+	0.000	0.003	0.019	0.000	0.992	0.150	6.608	0.000	1.913	0.334
	Cluster_82	Oscillospiraceae UCG-002	+	0.000	0.007	0.049	0.000	0.913	0.143	6.365	0.000	1.560	0.279
	Cluster_1	Bacteroides	-	0.003	0.300	-0.454	0.000	-0.301	0.066	-4.570	0.000	-0.546	0.104
	Cluster_109	Oscillibacter	-	0.003	0.001	-0.004	0.000	-0.409	0.081	-5.051	0.014	-0.695	0.170
	Cluster_403	[Ruminococcus] gnavus group	-	0.004	0.001	0.000	0.000	-0.676	0.124	-5.433	0.000	-1.440	0.279
	Cluster_8	Blautia	-	0.007	0.035	-0.086	0.000	-0.322	0.069	-4.646	0.000	-0.576	0.109
	Cluster_85	Lachnoclostridium	-	0.011	0.008	-0.014	0.000	-0.388	0.075	-5.202	0.000	-0.674	0.122
	Cluster_65	Tyzzerella	-	0.017	0.004	-0.011	0.000	-0.711	0.177	-4.019	0.042	-1.323	0.355
	Cluster_186	Ruminococcaceae UBA1819	-	0.022	0.000	-0.001	0.000	-0.386	0.077	-5.017	0.007	-0.782	0.178
	Mother born in France (no vs yes)												
	Cluster_174	Oscillospiraceae (unknown genus)	-	0.003	0.001	-0.004	0.002	-0.583	0.135	-4.331	0.032	-0.285	0.138
	otu.id	Family											
	Gestational age (<37 weeks of GA vs >= 37 weeks of GA)												
	Cluster_117	Butyricicoccaceae	-	0.000	0.002	-0.010	0.000	-0.640	0.096	-6.658	0.000	-1.009	0.174
	Cluster_299	Erysipelatoclostridiaceae	-	0.000	0.000	-0.003	0.000	-0.665	0.088	-7.550	0.000	-1.590	0.254
	Household siblings (At least 1 vs none)												
Family level	Cluster_62	Marinifilaceae	+	0.000	0.005	0.021	0.009	0.355	0.127	2.801	0.006	0.956	0.243
	Cluster_68	Christensenellaceae	+	0.000	0.008	0.070	0.000	0.718	0.142	5.072	0.000	1.449	0.267
	Cluster_79	Barnesiellaceae	+	0.000	0.008	0.036	0.004	0.533	0.172	3.104	0.025	1.241	0.345
	Cluster_1	Bacteroidaceae	-	0.003	0.300	-0.454	0.000	-0.491	0.067	-7.300	0.000	-0.550	0.104
	Cluster_389	Eggerthellaceae	-	0.012	0.000	-0.001	0.000	-0.573	0.078	-7.323	0.003	-0.805	0.181

LDM: mean.freq= the mean relative abundance of taxa in the tested category, beta= effect size. ANCOM-BC: beta= coefficient obtained from the ANCOM-BC log linear (natural log) model (log-transformed change in abundance), se = standard error of the beta, W = test statistic (beta/se). ALDEx2: beta= per-feature effect size, se= standard error of the beta. Direction: - = less abundant taxa in the category compared to the reference, + = more abundant taxa in the category compared to the reference. adj.pvalue = False discovery rate (DFR) adjusted p-value.

The relative abundance of the *Bifidobacterium* genus was significantly different between preterm and full-term children according to both LDM and ANCOM-BC methods (FDR p = 0.037 and FDR p = 0.01 respectively). However, no difference was observed according to ALDEx2 method (FDR p = 0.68).

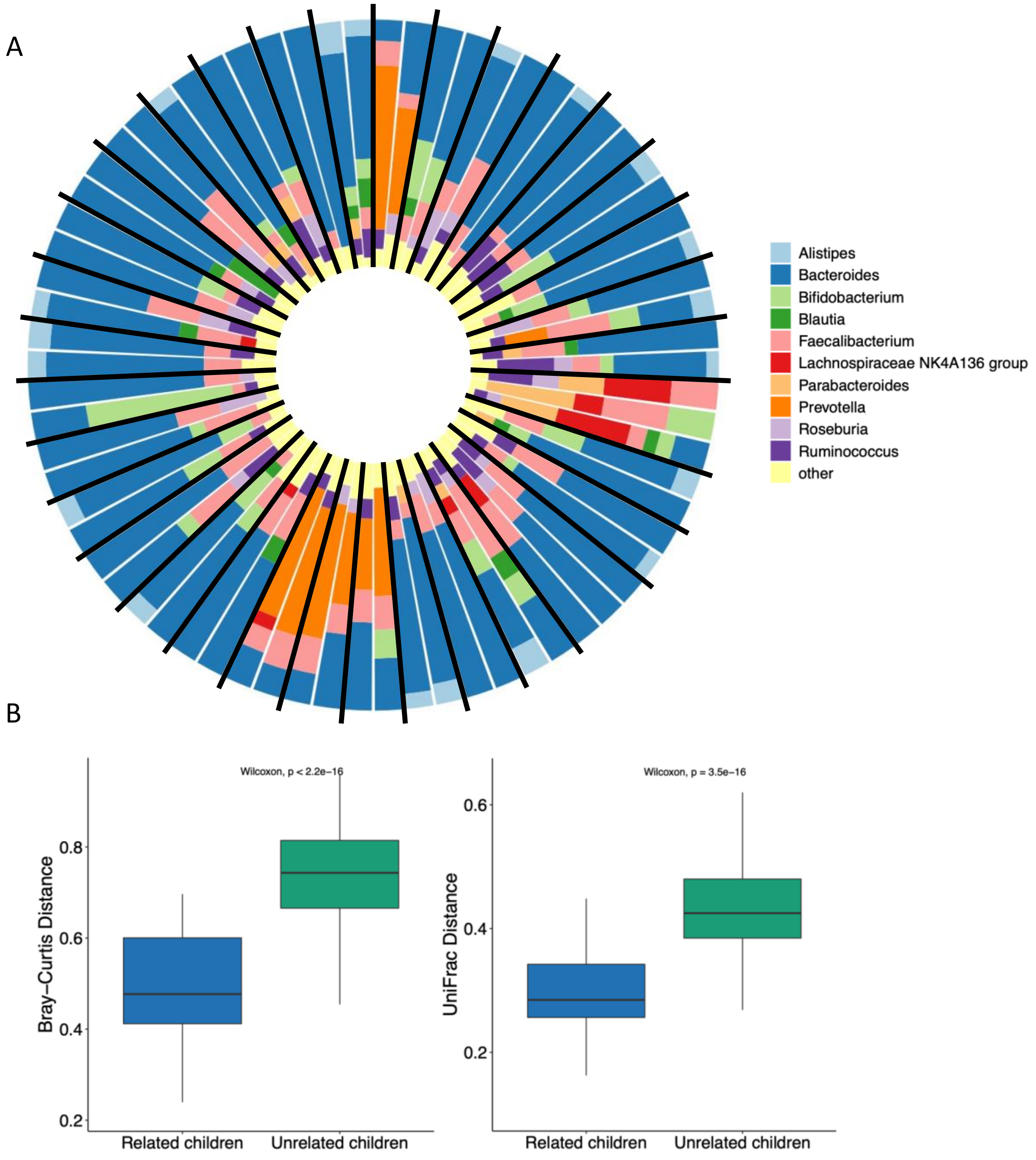

Figure S1. Relatedness of gut microbiota at 3.5 years of age in related EPIPAGE2 children. A Barplots showing the relative abundance of the top 10 genus in the gut microbiota of the 36 EPIPAGE 2 children that have a related sibling in the cohort. Bars between two dark lines referred to a set of related children (twins or triplets). **B** Pairwise beta diversity distances between related and unrelated children according to both Bray-Curtis and UniFrac distances. Significant differences were assessed by Wilcoxon rank sum test.

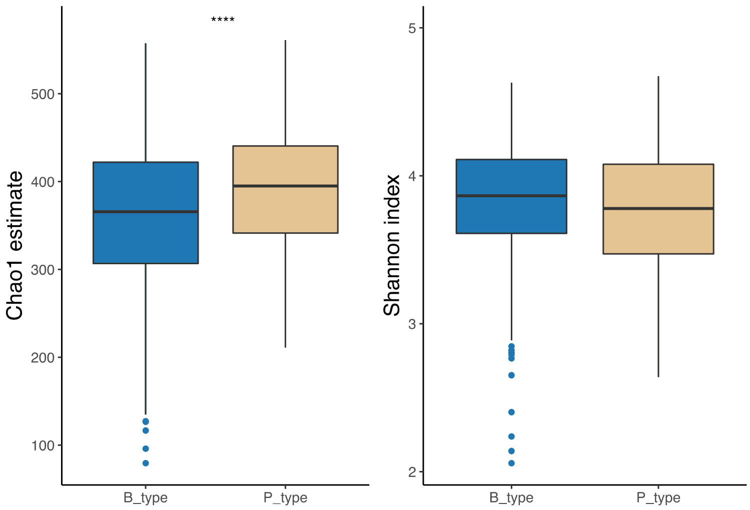

Figure S2. Alpha diversity across enterotypes. Boxplot of alpha diversity assessed by Chao1 estimate (left) and Shannon index (right) based on the OTU taxonomic profile between B_type and P_type enterotypes. (****$p \leq 0.0001$). The boxplots show the smallest and largest values, 25% and 75% quartiles, the median, and outliers.
